# "Cullin 4 makes its mark on chromatin"

**DOI:** 10.1186/1747-1028-1-14

**Published:** 2006-07-10

**Authors:** Qian Dai, Hengbin Wang

**Affiliations:** 1 Department of Biochemistry and Molecular Genetics, University of Alabama at Birmingham, Kaul Human Genetics Building Room 402A, 720 South 20th Street, Birmingham, AL 35294, USA

## Abstract

Cullin 4 (Cul4), a member of the evolutionally conserved cullin protein family, serves as a scaffold to assemble multisubunit ubiquitin E3 ligase complexes. Cul4 interacts with the Ring finger-containing protein ROC1 through its C-terminal cullin domain and with substrate recruiting subunit(s) through its N-terminus. Previous studies have demonstrated that Cul4 E3 ligase ubiquitylates key regulators in cell cycle control and mediates their degradation through the proteasomal pathway, thus contributing to genome stability. Recent studies from several groups have revealed that Cul4 E3 ligase can target histones for ubiquitylation, and importantly, ubiquitylation of histones may facilitate the cellular response to DNA damage. Therefore, histone ubiquitylation by Cul4 E3 ligase constitutes a novel mechanism through which Cul4 regulates chromatin function and maintains genomic integrity. We outline these studies and suggest that histone ubiquitylation might play important roles in Cul4-regualted chromatin function including the cellular response to DNA damage and heterochromatin gene silencing.

## Background

The cullins are a group of evolutionarily conserved proteins that play important roles in many aspects of cell biology [1]. Cullin serves as a scaffold to assemble multisubunit ubiquitin E3 ligase complexes through its C-terminal interaction with the small Ring finger-containing protein ROC1 or its homolog ROC2/APC11 and its N-terminal interaction with a number of substrate adaptor proteins, which recognize and bind specific domains in the substrate [1-3]. Therefore, by serving as a bridge, Cullin E3 ligases bring the ubiquitin-conjugated E2 enzyme, through its interaction with the Ring finger domain, to substrates and mediate the isopeptide bond formation [4]. For example, Cullin 1 bridges F-box containing proteins to an E2 enzyme through the substrate adaptor protein SKP1, and Cullin 2- and Cullin 5 bridge SOCS proteins to E2 enzymes through the Elongin complex [1,2]. Some cullins, however, do not require substrate adaptor proteins. Cullin 3, for example, binds its substrate BTB domain-containing proteins directly [5,6]. Since there is a large number of F-box, SOCS and BTP-containing proteins in the genome, cullins can potentially regulate a broad range of physiological processes [1-3].

**Figure 1:**
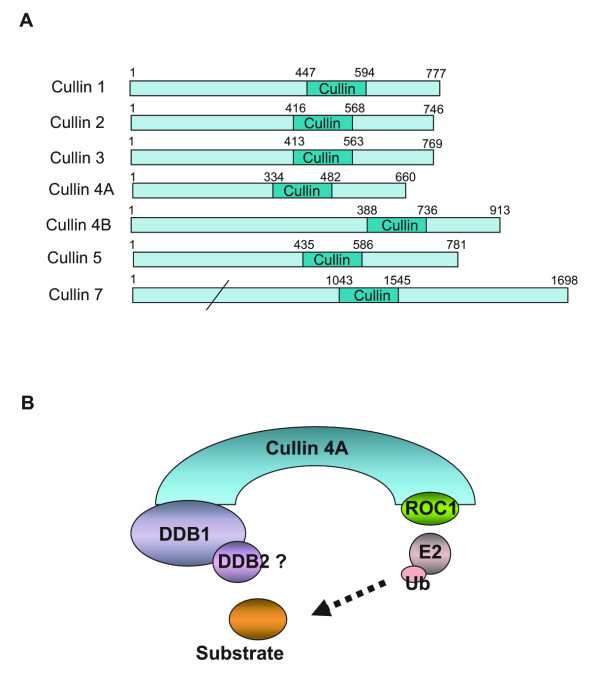
Cullin E3 ligase protein family in human cells. (A). Diagram of human Cullin 1, Cullin 2, Cullin 3, Cullin 4A, Cullin 4B, Cullin 5, and Cullin 7. The cullin domain of these proteins is shown. Numbers represent the amino acid number. (B). The modularity of Cullin 4A E3 ligase. Cullin 4A interacts with the Ring finger-containing protein ROC1 through its C-terminus Cullin domain. The N-terminus of Cullin 4A recruits substrates through DDB1-interacting proteins to ROC1, which interacts with ubiquitin conjugating enzymes (Ubc5) and mediates the ubiquitylation reaction.

There are seven closely related cullin family proteins in human cells: Cullin 1, Cullin 2, Cullin 3, Cullin 4A, Cullin 4B, Cullin 5, and Cullin 7 (Figure 1A) [1]. Each Cullin contains a ~150 amino acid Cullin domain at its carboxy-terminus, which mediates the interaction with the Ring finger motif in ROC1, or ROC2, or AOC11 [1]. The general structure of the scaffold is maintained in all cullins except for Cullin 4A (Cul4A), which contains a truncated N-terminus (Figure 1A) [7]. The truncation, which impairs its interaction with substrate adaptor subunits, is compensated by DDB1, a factor that was originally identified as a subunit of the DNA damage binding complex [8,9]. In this case, DDB1 functions as the subunit for substrate recruitment (Figure 1B) [8]. So far, several Cul4 E3 ligase complexes have been identified [10-12]. These complexes have similar compositions and have been implicated in cell cycle regulation, cell proliferation control, DNA damage checkpoint response, and etc [12,13].

## Discussion

### Cullin 4 and genomic integrity

Cul4 E3 ligase has been implicated in the maintenance of genomic integrity by promoting the ubiquitylation and subsequent degradation of key regulators in cell cycle regulation [12,13]. In *C. elegans*, mutation of Cul4 is associated with a massive DNA re-replication and cell cycle S-phase arrest [14]. Mutant cells accumulate high levels of CDT-1, a factor that is required for DNA replication during S-phase but has to be degraded at the end of S-phase to avoid DNA re-replication before the completion of cell division. The critical role of Cul4 in regulation of CDT-1 levels was demonstrated by an experiment showing that removing one genomic copy of cdt-1, which reduces the levels of CDT-1, suppresses the massive DNA re-replication in Cul4 mutant cells [14]. Moreover, Cul4 has also been implicated in governing genomic stability when cells face the challenge of genotoxic agents. In response to UV- or γ-irradiation, *Drosophila *S2 cells or HeLa cells degrade CDT-1 and arrest in cell cycle G1-phase. This G1-phase checkpoint blocks cell division before the damaged DNA is repaired and prevents transmission of the damaged DNA to daughter cells, thus ultimately contributing to genomic integrity. Knockdown of Cul4 in S2 cells or CUL4A and CUL4B in HeLa cells reduces the ability of these cells to degrade CDT-1 after genotoxic stress and impairs the cell cycle arrest. Therefore, Cul4 E3 ligase could govern genomic integrity by regulating the levels of CDT-1 [12]. Moreover, Cul4 E3 ligase has been implicated in the regulation of cell proliferation by the ubiquitylation and degradation of the oncogene c-jun and other nuclear factors, indicating a potential link between Cul4 and tumorigenesis [7,11]. Consistently, the CUL4A gene is amplified or overexpressed in a portion of breast and liver tumors [15,16].

In addition to checkpoint responses, Cul4 might regulate the repair of damaged DNA directly. CUL4A was identified as a component of two similar E3 ligase complexes that function in general and transcriptional-coupled nucleotide excision repair (NER) pathway, respectively [10]. NER is an essential cellular defense mechanism that is responsible for removing a variety of helix-distorting DNA damages such as UV-induced CPD and 6-4 PPs [17]. Importantly, UV irradiation causes the dissociation of inhibitory subunits from the E3 ligase complex and targets it to the damaged chromatin [10,18,19]. Two factors, DDB2 and XPC, which function as damage detectors in the early step of the NER pathway, were indicated as substrates for the Cul4A E3 ligase [19,20]. It was found that ubiquitylation of XPC and DDB2 by Cul4A E3 ligase regulates their binding activities toward the damaged DNA in opposite directions. Ubiquitylation of DDB2 causes the release of this factor (possibly the whole Cul4A E3 ligase complex) from the damaged DNA while ubiquitylation of XPC facilitates its binding to the damaged DNA [20]. Therefore, ubiquitylation of DDB2 and XPC by Cul4A E3 ligase constitutes a mechanism that handovers the damage signal from DDB2 to XPC, the latter of which initiates the assembly of downstream NER machinery at the damage foci [20].

### Cullin4 and histone ubiquitylation

In eukaryotic cells, the genomic DNA is complexed with histones to form chromatin, which serves as templates for transcription, replication, recombination, and repair [21,22]. Posttranslational modifications of histones at their N-terminal and C-terminal tails play a pivotal role in regulating the accessibility of DNA as well as the recruitment of modular proteins [23]. Histone modifications including phosphorylation, acetylation, and methylation have been implicated in the cellular response to DNA damage [24]. Moreover, chromatin remodeling activity has also been implicated in the damage repair process [25].

The first link between Cul4 E3 ligase and chromatin came from studies of the cellular response to UV irradiation. Prior to UV irradiation, Cul4A E3 ligase was found in the soluble nuclear extract fraction and its ubiquitin ligase activity was masked by an association with the subunits of COP9 signalsome (CSN), which are negative regulators of the ubiquitin ligase activity. Upon UV irradiation, Cul4A E3 ligase dissociates from the subunits of CSN and binds to chromatin tightly. Importantly, core histones including H3, H4, H2A, and H2B were copurified with Cul4A E3 ligase. This study indicated that Cul4A E3 ligase might target chromatin directly, although it is not clear whether histones are its physiological substrates [10].

A direct link between Cul4 E3 ligase and chromatin came from an unbiased search for activities in cell extracts that could ubiquitylate histones [26,27]. One activity that ubiquitylates all core histones turned out to be a Cul4 E3 ligase, the CUL4-DDB-ROC1 complex. Although this complex has compositions similar to the previously identified Cul4A E3 ligases [8,10-12], it bears unique features. First, this complex contains both Cul4A and Cul4B. Second, this complex contains both DDB1 and DDB2. Third, this complex is devoid of subunits of CSN, the negative regulators for ubiquitin ligase activity. Whether these unique features confer CUL4-DDB-ROC1 to ubiquitylate nucleosomes or whether the previously reported Cul4A E3 ligases also have activity toward nucleosomes needs to be investigated in the future.

To determine the role of CUL4-DDB-ROC1 in histone ubiquitylation *in vivo*, RNAi-mediated knockdown experiments were carried out. Knockdown of Cul4A or Cul4B in HeLa cells significantly reduces the levels of H3 and H4 ubiquitylation but has little effects on H2A and H2B ubiquitylation, indicating that the CUL4-DDB-ROC1 complex is a *bone fide *histone ubiquitin ligase, mainly for histones H3 and H4. The failure to detect changes in H2A and H2B ubiquitylation in CUL4 knockdown cells is consistent with previous reports that H2A and H2B are ubiquitylated by RNF2 and RNF20/RNF40, respectively [27-30]. It is also possibly that the high basal levels of H2A and H2B ubiquitylation prevent the detection CUL4-DDB-ROC1-mediated H2A and H2B ubiquitylation. Intriguingly, the levels of H3 and H4 ubiquitylation are induced by UV irradiation. The ubiquitylation levels increase quickly after UV irradiation (detectable at ~10 min), reach peaks between 1–2 hrs, decrease after 4 hrs and return to the normal level after 8 hrs. This dynamic change indicates that histone ubiquitylation by CUL4-DDB-ROC1 may participate in an early step of the damage repair process. To test this possibility, the recruitment of the damage recognition protein XPC to the damaged foci was examined in control and Cul4A knockdown cells. Immunostaining revealed that in control cells XPC started to relocate to the damage foci immediately following UV irradiation (0 min) and completely colocalized with the damage foci 30 min after the irradiation. However, colocalization of XPC with the damage foci was not observed under the same conditions in the Cul4A knockdown cells. This indicates that Cul4A, which is required for H3 and H4 ubiquitylation, plays an important role in the recruitment of repair proteins to the damage foci. Consistently, Cul4A knockdown has reduced the ability of cells to repair UV-induced lesions. Furthermore, histone ubiquitylation by CUL4-DDB-ROC1 weakens the interaction between histones and DNA. Based on these results, a model was proposed for the involvement of CUL4-DDB-ROC1-mediated histone ubiquitylation in the cellular response to UV damage. Upon UV irradiation, CUL4-DDB-ROC1 is recruited to the damage foci and ubiquitylates histones around the lesion, which causes histone eviction from the damaged nucleosomes and exposes the damaged DNA to repair proteins [26].

The link between Cul4 E3 ligase and histone ubiquitylation was also reported by Kapetanaki et al [18]. Based on the observations that Cul4A E3 ligase is recruited to damaged foci upon UV irradiation [18,19], the authors seek to identify the physiological substrates during this process. Ubiquitylated H2A represents a good candidate, as H2A is predominately ubiquitylated up to 10% in mammalian cells [31]. An investigation of the dynamic changes in H2A ubiquitylation in response to UV irradiation revealed an interesting finding. In normal lymphoblastoid cells, the levels of H2A ubiquitylation drop dramatically upon irradiation (detectable at 0 min), begin to recover at 30 min, and restore to normal levels at 2 hrs. However, in the DDB2 mutant XP-E cells, the recovery of uH2A was not observed. Based on these experiments, the authors suggested that deubiquitylation of H2A may serve as a stress sensor in the initial phase of UV irradiation and that the Cul4 E3 ligase is responsible for restoring the normal levels of H2A ubiquitylation in the later phase of repair. Moreover, this study identified DDB2 as a key subunit for histone H2A ubiquitylation, which is in conflict with previous reports that Ring2 ubiquitylates histone H2A *in vivo *[27,32]. The changes in H3 and H4 ubiquitylation in response to UV irradiation were not observed in this study, possibly due to the low levels of H3 and H4 ubiquitylation and the lack of available antibodies against ubiquitylated histone H3 and H4 [26].

The dynamic changes of H2A ubiquitylation in response to UV irradiation were also noted by Bergink et al., however, different profiles were observed for H2A ubiquitylation after UV irradiation [33]. Employing single living cell imaging technology, the authors found that local UV irradiation caused an enrichment of ubiquitin conjugate at the UV damage foci. The ubiquitin conjugate was identified as ubiquitylated H2A, as it could be recognized by the ubiquitylated H2A-specific antibody [34]. A link between H2A ubiquitylation and cellular damage repair was uncovered by the finding that the UV-induced enrichment of ubiquitylated H2A was absent in the NER-deficient XPA, XPC, XPG, and XPF cells but remains intact in the XPV cells, which are not defective in NER pathway but carry a mutant DNA polymerase η. This experiment established the dependence of H2A ubiquitylation on the NER pathway and suggests that H2A ubiquitylation functions in late stages of the repair process e.g., after incision of the damaged strand. Consistent with the notion that Ring2 is the H2A-specfiic ubiquitin ligase [27,32], Ring2 was found to be required for H2A ubiquitylation during this process. Therefore, in contrast to Kapetanaki's reports [18], an increase of H2A ubiquitylation in response to UV irradiation was observed in this study.

## Conclusion

**Figure 2:**
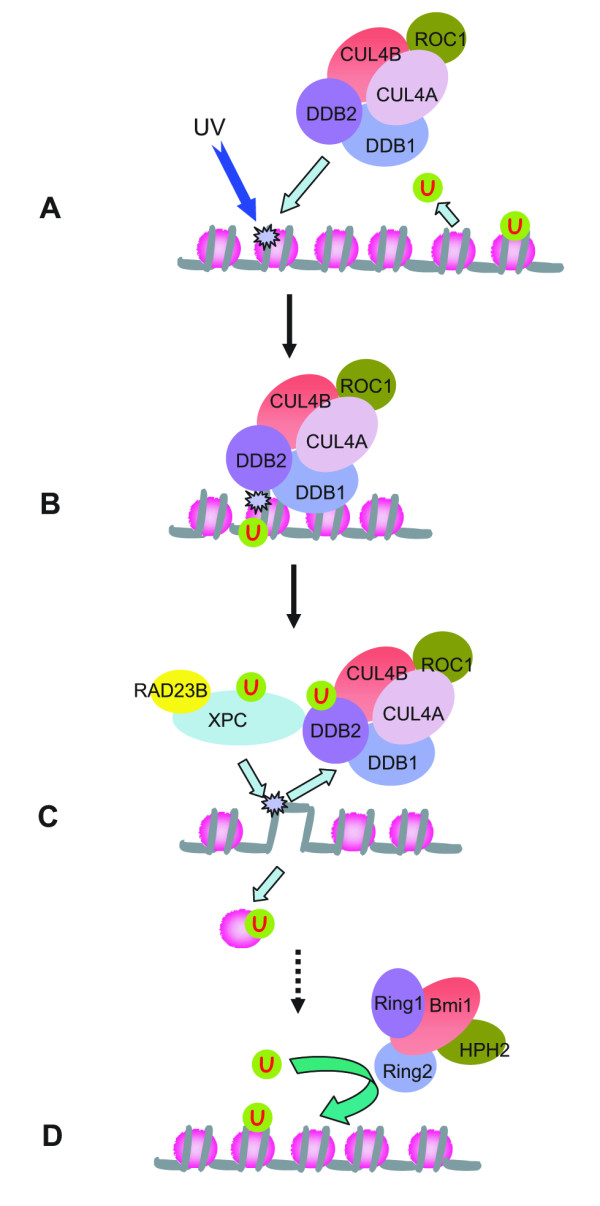
A model depicting the involvement of histone ubiquitylation in the cellular response to UV damage. (A). Upon UV irradiation, the histone ubiquitin ligase CUL4-DDB-ROC1 is recruited to the damaged chromatin through DDB2. Meanwhile, the ubiquitin moiety is cleaved from ubiquitylated H2A when cells sense the DNA damage. (B). CUL4-DDB-ROC1 ubiquitylates histones around the lesion, using ubiquitin released from H2A deubiquitylation. (C). Histone ubiquitylation by CUL4-DDB-ROC1 causes histone eviction from the damaged nucleosome. Meanwhile, Cul4 E3 ligase ubiquitylates DDB2 and XPC, resulting in the release of Cul4 E3 ligase from the damaged DNA and facilitating the binding of XPC to the damaged DNA. (D). After the repair processes, histone H2A around the DNA lesion is ubiquitylated in a Ring2 or Cul4 dependent manner.

The three recent studies point to the intriguing link between histone ubiquitylation (H3, H4, and H2A) and the cellular response to DNA damage, although a number of discrepancies were found in detailed results [18,26,35]. We believe that these discrepancies are due to the experimental approaches employed in each study, which focused on different aspects of histone ubiquitylation and DNA damage responses, and therefore, only caught portions of the whole picture. Based on these and previous studies, we propose a sequential model for the involvement of histone ubiquitylation in the cellular response to DNA damage (Figure 2).

Upon UV irradiation, CUL4-DDB-ROC1 is recruited to the damage foci through the binding of DDB2 to the damaged DNA [18,19]. Meanwhile, the ubiquitin moiety is cleaved from the ubiquitylated H2A [18] when cells sense the genotoxic stress [36,37] (Figure 2A, these responses could be detected at 0 min after UV irradiation). CUL4-DDB-ROC1 ubiquitylates histones around the DNA lesions (mainly H3 and H4, detectable as early as 5~10 min after UV irradiation) [26]. The ubiquitin for the reaction may come from the deubiquityaltion of H2A [18] (Figure 2B). Histone ubiquitylation by CUL4-DDB-ROC1 weakens their interaction with DNA and causes histone eviction from the damaged nucleosomes [26]. Meanwhile, ubiquitylation of DDB2 and XPC by Cul4 E3 ligase changes their damaged DNA binding activity, causing the release of Cul4 E3 ligase and facilitating the binding of XPC to the damaged DNA [20] (Figure 2C). Finally, after the NER process, the restored nucleosomes around the lesions (which can be up to 10–30 kbp long) [35] are ubiquitylated at histone H2A by the Ring2 or Cul4 histone ubiquitin ligases (Figure 2D, detectable 30 min after UV irradiation) [18,35]. The functional significance of H2A ubiquitylation after NER is not clear. One possibility is to facilitate the chromatin fiber restoring its original configuration, however, this notion is mainly a speculation.

Based on this model, Cul4 E3 ligase plays a central role in the cellular response to UV damage by coordinately ubiquitylating a number of different substrates (Figure 2). To validate this model, the dynamic association of ubiquitylated histones (H3/H4 vs H2A) and protein factors with the damaged DNA will have to be monitored at high temporal resolution after UV irradiation. However, UV-induced DNA lesions are not sequence-specific, so the standard chromatin immunoprecipitate protocol has to be modified.

The unveiling of the link between Cul4 E3 ligase and histone ubiquitylation sheds light on the mechanism of Cul4-mediated heterochromatin assembly [38]. Recently, Cul4 was found to interact with the heterochromatin protein Rik1 (share extensive homology with DDB1) and the H3K9 methyltransferase Clr4 in fission yeast [39-41]. Mutation of Cul4 or expression of the dominant negative Cul4 results in dysfunction of all heterochromatin regions. Does Cul4-mediated histone ubiquitylation play a role in this process? H2B ubiquitylation in budding yeast could regulate the activity of methyltransferases Dot1 and Set1, allowing them to di- and tri-methylate lysine residues [42]. Is there a similar mechanism operating between Clr4 and Cul4-mediated histone ubiquitylation? Further investigations will clarify these issues.

## Competing interests

The author(s) declare that they have no competing interests.

## Authors' contributions

QD contributed to the initial draft of the manuscript. HBW wrote the manuscript. All authors read and approved the finial manuscript.
